# SALM5 trans-synaptically interacts with LAR-RPTPs in a splicing-dependent manner to regulate synapse development

**DOI:** 10.1038/srep26676

**Published:** 2016-05-26

**Authors:** Yeonsoo Choi, Jungyong Nam, Daniel J. Whitcomb, Yoo Sung Song, Doyoun Kim, Sangmin Jeon, Ji Won Um, Seong-Gyu Lee, Jooyeon Woo, Seok-Kyu Kwon, Yan Li, Won Mah, Ho Min Kim, Jaewon Ko, Kwangwook Cho, Eunjoon Kim

**Affiliations:** 1 Department of Biological Sciences, Korea Advanced Institute for Science and Technology (KAIST), Daejeon 305-701, Korea; 2 Henry Wellcome Laboratories for Integrative Neuroscience and Endocrinology, School of Clinical Sciences, Faculty of Health Sciences, University of Bristol, Whitson Street, Bristol BS1 3NY, United Kingdom; 3 Department of Nuclear Medicine, Seoul National University Bundang Hospital, Gyeonggi-do, 463–707, Korea; 4 Center for Synaptic Brain Dysfunctions, Institute for Basic Science (IBS), Daejeon 305-701, Korea; 5 Department of Biochemistry, College of Life Science and Biotechnology, Yonsei University, Seoul 120-749, Korea; 6 Department of Physiology and BK21 PLUS Project for Medical Science, Yonsei University College of Medicine, Seoul 120-752, Korea; 7 Department of Anatomy and Neurobiology, School of Dentistry, Kyungpook National University, Daegu, Korea; 8 Graduate School of Medical Science and Engineering, KAIST, Daejeon 305-701, Korea; 9 Centre for Synaptic Plasticity, University of Bristol, Bristol BS1 3NY, United Kingdom

## Abstract

Synaptogenic adhesion molecules play critical roles in synapse formation. SALM5/Lrfn5, a SALM/Lrfn family adhesion molecule implicated in autism spectrum disorders (ASDs) and schizophrenia, induces presynaptic differentiation in contacting axons, but its presynaptic ligand remains unknown. We found that SALM5 interacts with the Ig domains of LAR family receptor protein tyrosine phosphatases (LAR-RPTPs; LAR, PTPδ, and PTPσ). These interactions are strongly inhibited by the splice insert B in the Ig domain region of LAR-RPTPs, and mediate SALM5-dependent presynaptic differentiation in contacting axons. In addition, SALM5 regulates AMPA receptor-mediated synaptic transmission through mechanisms involving the interaction of postsynaptic SALM5 with presynaptic LAR-RPTPs. These results suggest that postsynaptic SALM5 promotes synapse development by trans-synaptically interacting with presynaptic LAR-RPTPs and is important for the regulation of excitatory synaptic strength.

Synaptic adhesion molecules with synaptogenic activity play important roles in several steps of synapse development, including the initial contact between axons and dendrites, and formation and maturation of early synapses^1^,^2^,^3^,^4^,^5^,^6^,^7^,^8^,^9^,^10^,^11^,^12^,^13^.

The SALM (for synaptic adhesion-like molecules; also known as Lrfn for leucine-rich repeat and fibronectin type III domain containing) family of adhesion molecules contains five known members: SALM1/Lrfn2, SALM2/Lrfn1, SALM3/Lrfn4, SALM4/Lrfn3, and SALM5/Lrfn5^13^,^14^,^15^,^16^. SALMs share a similar domain structure, containing leucine-rich repeats (LRRs), an immunoglobulin (Ig) domain, and a fibronectin III (FNIII) domain, followed by a transmembrane domain and a C-terminal PDZ-binding motif. This motif, known to bind the postsynaptic scaffolding PDZ protein PSD-95, is present in SALMs 1–3, but not in SALMs 4–5, suggesting that SALMs have diverse functions. Further supporting the functional diversity of SALMs, SALM3 and SALM5 but not other SALMs induce presynaptic differentiation in contacting axons^17^. Notably, SALM4 and SALM5, but not other SALMs, display homophilic and trans-cellular adhesion^18^. This suggests that SALMs have both pre- and postsynaptic functions, and that trans-synaptic, homophilic SALM5 adhesion may play important roles.

SALM5/Lrfn5 has been associated with severe progressive autism in which expression levels of SALM5 are markedly reduced (by ~90%) due to a balanced chromosomal translocation^19^,^20^,^21^,^22^,^23^. In addition, an inherited copy number variation of SALM5 has been found in familial schizophrenia^24^,^25^. Therefore, SALM5-dependent presynaptic induction may play critical roles in brain development and function. However, the presynaptic ligands that mediate SALM5-dependent presynaptic induction have remained elusive. In addition, it is unclear whether the main function of SALM5, which lacks PSD-95 binding, is to induce presynaptic differentiation, minimally contributing to postsynaptic functions.

LAR (for leukocyte common antigen-related) is a family of receptor protein tyrosine phosphatases (LAR-RPTPs) with three known members: LAR/PTPRF, PTPδ/PTPRD, and PTPσ/PTPRS. Early studies on Drosophila LAR implicated the protein in the regulation of axon guidance and synaptic development^26^,^27^. In mammals, LAR-RPTPs have been shown to regulate dendrite and excitatory synapse development and maintenance^28^,^29^. LAR-RPTPs interact trans-synaptically with several postsynaptic adhesion molecules, including NGL-3, TrkC, IL1RAPL1, IL1RAcP, Slitrks, and SALM3 to promote synapse development^30^,^31^,^32^,^33^,^34^,^35^,^36^,^37^,^38^,^39^, and in cis manner with presynaptic glypicans and netrin-G1^40^,^41^. Supporting their significant in vivo functions, mice lacking LAR, PTPδ, or PTPσ exhibit a variety of phenotypes, including reduced food intake and survival, endocrine defects, reduced neuronal growth and regeneration, altered synaptic plasticity, impaired learning and memory, and hyperactivity^42^,^43^,^44^,^45^,^46^,^47^,^48^,^49^,^50^. In humans, PTPδ has been implicated in ASDs, ADHD, bipolar disorder, and restless leg syndrome^51^,^52^,^53^,^54^,^55^, although the underlying mechanisms are largely unknown.

Here, we identified LAR-RPTPs as novel and splicing-dependent presynaptic ligands for SALM5, and demonstrate that they mediate SALM5-dependent presynaptic differentiation. In addition, we found that SALM5 maintains AMPA receptor (AMPAR)-mediated excitatory synaptic transmission through mechanisms involving the interaction of SALM5 with LAR-RPTPs.

## Results

### SALM5 interacts with LAR-RPTPs

In order to identify a presynaptic ligand for SALM5, we performed cell aggregation assays in which one group of L cells expressing SALM5 was mixed with another group of L cells expressing candidate adhesion molecules (Supplementary Fig. 1). We found that SALM5-expressing cells coaggregated with cells expressing all three LAR-RPTPs (LAR, PTPδ, and PTPσ). The reported transcellular, homophilic adhesion for SALM4 and SALM5^18^ was not be observed in the form of green-cell aggregates in our assays, likely due to that we used floating L cells with a low adhesive background to make a high-stringency condition, whereas previous assays employed HeLa cells or hippocampal neurons attached to extracellular substratum. In addition, we could not observe self-aggregations of LAR-RPTPs, as also shown previously for PTPσ^56^.

**Figure 1:**
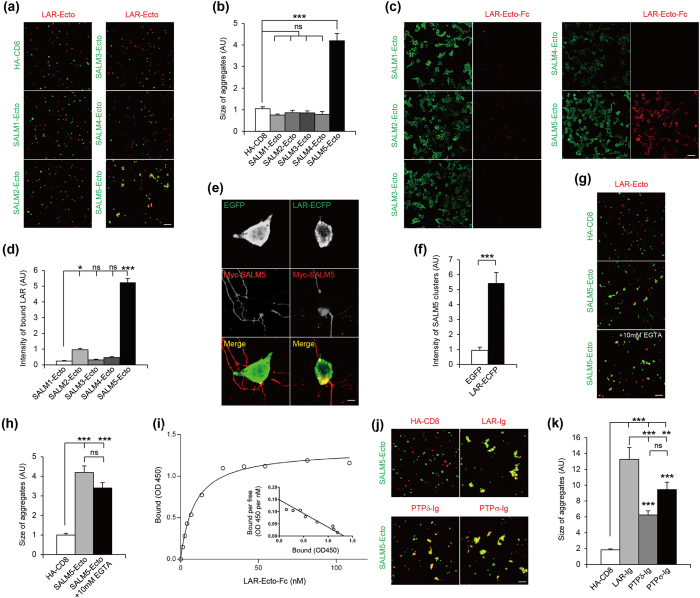
SALM5, but not other SALMs, interacts with LAR-RPTPs. (**a**) SALM5, but not other SALMs, interacts with LAR in cell aggregation assays. A group of L cells doubly transfected with pDisplay constructs containing the ectodomains of SALMs (SALMs-Ecto-pDis) and EGFP was mixed with another group of L cells cotransfected with LAR-Ecto-pDis (meA^−^; meB^−^) and DsRed2 for cell aggregation. Scale bar, 100 μm. (**b**) Quantification of the results in (A). Mean ± s.e.m. *n* = 10 fields of view, ***p < 0.001, ANOVA-Tukey’s test. (**c**) SALM5 binds soluble LAR. HEK293T cells expressing SALMs-Ecto-pDis were incubated with LAR-Ecto-Fc (meA^−^ and meB^−^). Scale bar, 50 μm. (**d**) Quantification of the results in (**c**). *n* = 15 fields of view, *p < 0.05, ***p < 0.001, ANOVA-Tukey’s test. (**e**) LAR-expressing HEK293T cells induce SALM5 clustering in contacting neurites of cocultured neurons. HEK293T cells expressing LAR (C-terminal ECFP tag) were cocultured with hippocampal neurons (DIV 14–17) transfected with SALM5 (N-terminal Myc tag; DIV 12–14). The large, discontinued SALM5 clusters are likely because they are different neurites and exogenously expressed proteins. Scale bar, 10 μm. (**f**) Quantification of the results in (**e**). *n* = 10 cells for EGFP and 7 for LAR-ECFP, ***p < 0.001, Student’s t-test. (**g**) Calcium-insensitive adhesion between SALM5 and LAR. Two groups of L cells expressing SALM5-Ecto-pDis and LAR-Ecto-pDis were mixed for cell aggregation in the presence of 10 mM EGTA, a calcium chelator. Scale bar, 100 μm. (**h**) Quantification of the results in (**g**). *n* = 10, ***p < 0.001, ns, not significant, ANOVA-Tukey’s test. (**i**) Apparent affinity of the SALM5–LAR interaction measured by adding increasing amounts of LAR-Ecto-Fc to SALM5 (untagged)-expressing HEK293T cells. Inset, Scatchard plot analysis. (**j**) SALM5 interacts with PTPδ and PTPσ in cell aggregation assays. Note that we used the Ig domains of LAR-RPTPs because they mediate the SALM5 binding (see below for details). Scale bar, 100 μm. (**k**) Quantification of the results in (**j**). *n* = 10, ***p < 0.001, ANOVA-Tukey’s test.

Other SALMs (SALM1, SALM2, SALM3, and SALM4) did not interact with LAR in cell aggregation assays (Fig. 1a,b). Soluble LAR, the ectodomain of LAR fused to the Fc domain of human Ig (LAR-Ecto-Fc), bound strongly to SALM5-expressing HEK293T cells, but much more weakly to SALM2 and not to other SALMs (Fig. 1c,d). In addition, LAR expressed in HEK293T cells induced clustering of SALM5 as well as Shank2, a postsynaptic scaffold, in the neurites of cocultured hippocampal neurons (Fig. 1e,f; Supplementary Fig. 2).

The interaction between SALM5 and LAR was calcium-insensitive, as shown by the absence of an effect of the calcium chelator EGTA on the interaction (Fig. 1g,h). In order to determine the apparent binding affinity of the SALM5-LAR interaction, we incubated SALM5-expressing HEK293T cells with increasing amounts of LAR-Ecto-Fc. The K_d_ value of the SALM5-LAR interaction calculated by Scatchard analysis was 8.96 ± 0.92 nM (Fig. 1i). PTPδ and PTPσ also interacted with SALM5 in cell aggregation assays (Fig. 1j,k).

### The LRR domain of SALM5 interacts with Ig domains of LAR

**Figure 2:**
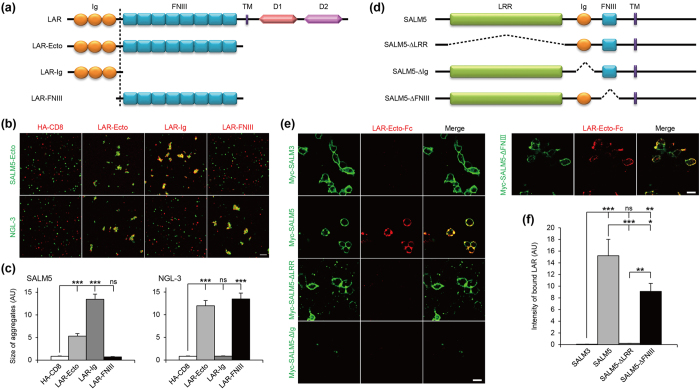
The LRR domain of SALM5 interacts with Ig domains of LAR. (**a**) Deletion variants of LAR used. TM, transmembrane domain; D1 and D2, tyrosine phosphatase domains. (**b**) The Ig domains of LAR mediate SALM5 binding. L cells expressing SALM5-Ecto-pDis, NGL-3 (untagged full-length, a positive control for LAR-FNIII), or HA-CD8 (a negative control), were mixed with other L cells expressing full-length or deletion variants of LAR-pDis for cell aggregation. Scale bar, 100 μm. (**c**) Quantification of the results in (**b**). Mean ± s.e.m. *n* = 10 fields of view, ***p < 0.001, ANOVA-Tukey’s test. (**d**) Deletion variants of SALM5 generated from full-length Myc-SALM5. (**e**) SALM5-ΔFNIII containing LRR + Ig domains binds LAR, whereas SALM5-ΔLRR, which lacks the LRR domain, fails to bind LAR. HEK293T cells expressing SALM5 deletion variants were incubated with LAR-Ecto-Fc, followed staining for surface Myc (SALM5) and human-Ig (LAR-Ecto-Fc). Note that SALM5-ΔIg fails to reach the cell surface. (**f**) Quantification of the results in (**e**), by normalizing bound LAR to surface Myc signals. *n* = 10 fields of view, ***p < 0.001, ANOVA.

In order to determine the minimal domains involved in the SALM5-LAR interaction, we generated deletion variants of SALM5 and LAR. A LAR variant containing the three Ig domains only (LAR-Ig) interacted with SALM5 in cell aggregation assays (Fig. 2a–c). In contrast, another region of LAR containing eight FNIII domains (LAR-FNIII) did not interact with SALM5, although it did interact with NGL-3 (Fig. 2a–c), a positive control known to interact with the first two FNIII domains of LAR, PTPδ, and PTPσ^30^,^32^. In soluble protein binding assays, purified LAR-Ig (Ig1-3 domains only) fused to Fc (LAR-Ig-Fc) bound to cells expressing SALM5 but not to those expressing SALM3 or NGL-3, whereas LAR-FN12-Fc (first two FNIII domains only) bound to NGL-3 but not to SALM3 or SALM5 (Supplementary Fig. 3a–d). These results suggest that the Ig domains of LAR are sufficient to mediate SALM5 binding, and indicate that two distinct domains of LAR mediate interactions with SALM5 (Ig domains) and NGL-3 (FNIII domains).

The deletion variants of SALM5 expressed in heterologous cells exhibited the predicted sizes and showed comparable surface expression, although some variants (ΔIg and ΔFNIII) displayed lowered expression levels, likely due to protein degradation (Fig. 2d–f and Supplementary Fig. 3e,f). When tested for LAR binding in soluble LAR-binding assays, a mutant SALM5 that lacked the FNIII domain but contained intact LRRs + Ig domains (SALM5-ΔFNIII) showed partially reduced LAR-Ecto-Fc binding comparable to that of full-length SALM5. Another SALM5 variant that lacked the LRR domain but contained intact Ig + FNIII domains (SALM5-ΔLRR) did not interact with LAR. A mutant SALM5 that lacked the Ig domain (SALM5-ΔIg) did not reach the cell surface. These results suggest that the LRR domain of SALM5 is required for LAR binding, and that the LRR + Ig domains of SALM5 are sufficient for LAR binding. In addition, the FNIII domain may also partially contribute to the interaction.

### SALM5 directly interacts with LAR

**Figure 3:**
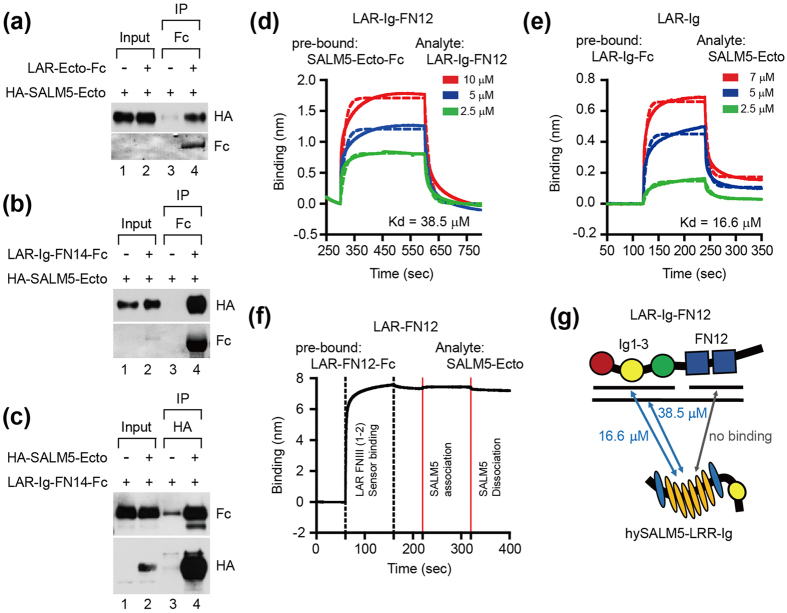
SALM5 directly interacts with LAR. (**a–c**) Coprecipitation between recombinant ectodomains of SALM5 and LAR. LAR ectodomain proteins (LAR-Ecto-Fc or LAR-IgFN14-Fc [Ig1-3 + FN1-4]) and HA-SALM5-Ecto secreted from transfected HEK293T cells into the supernatant were subjected to immunoprecipitation (IP) and immunoblot analysis. (**d–g**) Direct interaction between purified, soluble LAR and SALM5 fusion proteins, measured by the biolayer interferometry. Hybrid SALM5-Ecto proteins binds LAR-Ig-FN12 and LAR-Ig but not to LAR-FN12 fusion proteins (**d–f**), which is summarized in a schematic diagram (**g**). A hybrid SALM5 protein was used to increase protein expression levels (see Materials and methods for details). Note that the LAR constructs longer than the Ig domains only used here (LAR-FN12) and in the coimmunoprecipitation experiments (LAR-Ig-FN14) would not interfere with demonstrating the direction interaction of LAR with SALM5, as evident in Fig. 2a–c.

To obtain biochemical evidence for the interaction, we tried coimmunoprecipitation using soluble LAR fusion proteins (LAR-Ecto-Fc and LAR-Ig-FN14 [Ig + first four FNIII domains]) and soluble SALM5-Ecto, and found positive results (Fig. 3a–c).

Next, we tried a quantitative measurement of the direct interaction between LAR and SALM5 using soluble proteins and biolayer interferometry (BLI). SALM5-Ecto (containing the LRR + Ig domains but missing the following FNIII domain) displayed weak bindings to LAR-Ig-FN12 and LAR-Ig with the K_d_ values in the micromolar range (38.5 and 16.6 μM, respectively) but no binding to LAR-FN12 (Fig. 3d–g). These binding affinities are much lower than that for LAR-Ecto binding to SALM5 displayed on HEK293T cells (K_d_ of ~9 nM; Fig. 1i). It is possible that the SALM5 recombinant protein used in the current experiment may be in a suboptimal state for LAR binding, likely because we used a hybrid LRR version of SALM5 to increase protein expression levels (see Methods for further details). In addition, these in vitro experimental conditions may differ from the cellular context of the LAR binding assay (Fig. 1i).

### Splice inserts in LAR-RPTPs differentially regulate SALM5 binding

**Figure 4:**
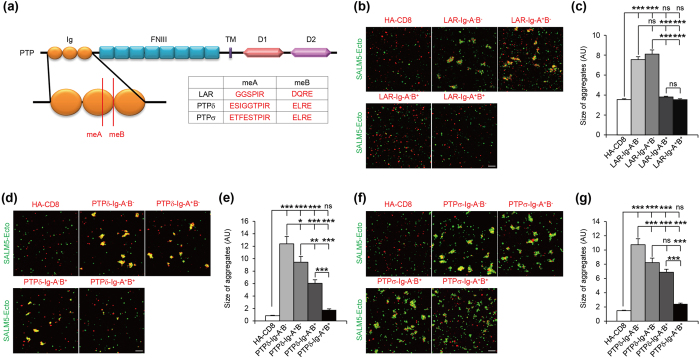
Splice inserts in the Ig domains of LAR, PTPδ, and PTPσ differentially regulate SALM5 binding. (**a**) Splice inserts (meA and meB) in the Ig domains of LAR-RPTPs. (**b**) The meB, but not meA, insert in LAR suppresses LAR binding to SALM5 in cell aggregation assays using the ectodomain of SALM5 and Ig domains of LAR (both in pDis). Scale bar, 100 μm. (**c**) Quantification of the results in (**b**). Mean ± s.e.m. *n* = 12 fields of view, ***p < 0.001, ANOVA-Tukey’s test. (**d**) Splice inserts (meA and meB) in PTPδ additively inhibit SALM5 binding. Scale bar, 100 μm. (**e**) Quantification of the results in (**d**). *n* = 10, *p < 0.05, **p < 0.01, ***p < 0.001, ANOVA-Tukey’s test. (**f**) Splice inserts (meA and meB) in PTPσ additively inhibit SALM5 binding. Scale bar, 100 μm. (**g**) Quantification of the results in (**f**). *n* = 10, ***p < 0.001, ANOVA-Tukey’s test.

The interaction of neurexins with neuroligins, LRRTM2, and Cbln1 + GluRδ2 are regulated by short peptide inserts in neurexins generated by alternative splicing^57^,^58^,^59^,^60^,^61^,^62^,^63^. Similarly, alternative splicing regulates LAR-RPTP interactions with TrkC, Slitrks, IL1RAPL1, IL1RAcP, and SALM3^33^,^35^,^36^,^39^. LAR-RPTPs contain short splice inserts in the Ig domains generated by mini exons (me) (Fig. 4a)^64^. Because the Ig domains of LAR mediated SALM5 binding, we tested whether the splice inserts in the Ig domains of LAR-RPTPs affected SALM5 binding.

We found that a splice insert of LAR from the first mini exon (meA; 6 amino acids) in the Ig domains had no additive effect on the interaction of LAR with SALM5 in cell aggregation assays using Ig domain-only LAR (Fig. 4a–c). The second mini exon (meB; 4 amino acids) in the Ig domains of LAR, however, significantly suppressed the interaction. PTPδ also contains two splice inserts (meA and meB) in the Ig domains (Fig. 4a). Partly dissimilar to the results from LAR, meA and meB in PTPδ additively suppressed PTPδ binding to SALM5 with a stronger effect being exerted by meB (Fig. 4d,e). Similar results were obtained for PTPσ binding to SALM5 (Fig. 4f,g).

Next, we used full-length LAR-RPTP proteins (not Ig domain only). We obtained similar results, as compared with those from Ig domain-only LAR-RPTPs, including that the meB insert strongly inhibits the interactions between SALM5 and LAR-RPTPs (Supplementary Fig. 4). However, a minor difference was that the meA insert in PTPσ, which inhibited the SALM5–PTPσ interaction (Ig domain-only PTPσ), slightly promoted the interaction (full-length PTPσ). This may be attributable to the different context of Ig domain-only and full-length constructs. Taken together, these results suggest that the meA and meB splice inserts in LAR-RPTPs differentially regulate SALM5 binding, and that the meB splice insert strongly inhibits the SALM5–LAR-RPTP interactions.

### Soluble LAR inhibits SALM5-dependent presynaptic differentiation

**Figure 5:**
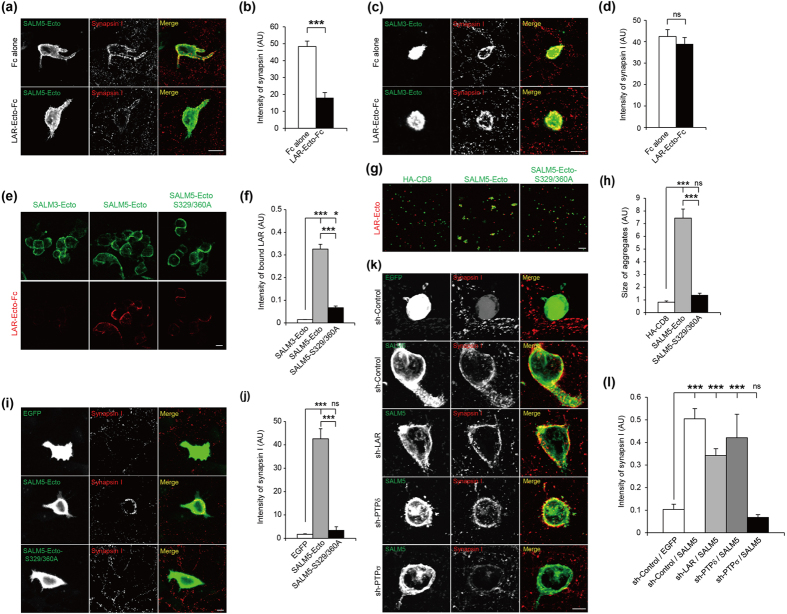
LAR-RPTPs mediate SALM5-dependent presynaptic differentiation. (**a**) Soluble LAR inhibits SALM5-dependent synapsin I clustering. SALM5-expressing HEK293T cells were cocultured with hippocampal neurons in the presence of LAR-Ecto-A^−^B^—^Fc, or Fc alone, followed by immunostaining for synapsin I and SALM5 (HA). Scale bar, 15 μm. (**b**) Quantification of the results in (**a**). Mean ± s.e.m. *n* = 39 fields of view for Fc alone and 33 for LAR-Ecto-Fc, ***p < 0.001, Student’s t-test. (**c**) Soluble LAR does not inhibit SALM3-induced synapsin I clustering. Scale bar, 15 μm. (**d**) Quantification of the results in (**c**). *n* = 26 for Fc alone and 17 for LAR-Ecto-Fc, ns, not significant, Student’s t-test. (**e**) A SALM5 mutant (S329/360A) shows reduced LAR binding. HEK293T cells expressing SALM5-Ecto-pDis, SALM5-Ecto-S329/360A-pDis, or SALM3-Ecto-pDis, were incubated with LAR-Ecto-Fc. Scale bar, 10 μm. (**f**) Quantification of the results in (**e**). *n* = 10, *p < 0.05, ***p < 0.001, ANOVA-Tukey’s test. (**g**) SALM5-S329/360A shows reduced binding to LAR. Scale bar, 100 μm. (**h**) Quantification of the results in (**g**). *n* = 10 fields of view, *p < 0.05, ***p < 0.001, ANOVA-Tukey’s test. (**i**) SALM5-S329/360A fails to induce synapsin I clustering in contacting axons. HEK293T cells expressing SALM5-Ecto-pDis, SALM5-Ecto-S329/360A-pDis, or EGFP alone, were cocultured with hippocampal neurons, followed by staining for synapsin I, SALM5 (HA), and EGFP. Scale bar, 10 μm. (**j**) Quantification of the results in (**i**). *n* = 13 cells for EGFP, 15 for SALM5-Ecto-pDis (WT and S329/360A), ***p < 0.001, ANOVA-Tukey’s test. (**k**) SALM5-dependent presynaptic synapsin I clustering is significantly reduced by knockdown of PTPσ but to a much lesser extent by LAR or PTPδ. Hippocampal neurons infected at DIV 3–4 with LAR-RPTP knockdown lentiviruses (sh-control/LAR/PTPδ/PTPσ) were cocultured for 3 days (DIV 10–13) with HEK293T cells expressing EGFP alone, or EGFP + SALM5-pDis, followed by staining for EGFP and synapsin I. Scale bar, 10 μm. (**l**) Quantification of the results in (**k**); synapsin I signals were normalized to EGFP fluorescence. n = 33 cells for EGFP/sh-Control, 22 for SALM5 + sh-Control/sh-PTPσ shRNA, and 19 for SALM5 + sh-LAR/sh-PTPδ, ***p < 0.001, ANOVA-Tukey’s test.

If LAR is a presynaptic ligand involved in SALM5-induced presynaptic differentiation, soluble LAR (LAR-Ecto-Fc), which would compete with endogenous LAR for SALM5 binding, should be able to inhibit SALM5-dependent presynaptic induction. Indeed, LAR-Ecto-Fc (meB-negative) added to SALM5-expressing HEK293T cells cocultured with hippocampal neurons was able to reduce SALM5-induced presynaptic protein (synapsin I) clustering in contacting axons, whereas Fc alone (a negative control) had no effect (Fig. 5a,b). In control experiments, LAR-Ecto-Fc (meB-negative) had no effect on SALM3-induced synapsin I clustering, similar to Fc alone (Fig. 5c,d), whereas another form of LAR-Ig-Fc (meB-positive) could inhibit SALM3-induced synapsin I clustering (Supplementary Fig. 5). These results suggest that LAR is important for presynaptic differentiation induced by SALM5.

### LAR-RPTPs mediate SALM5-dependent presynaptic differentiation

As an independent way to test the role of LAR in SALM5-dependent presynaptic differentiation, we attempted to generate a mutant SALM5 that lacked LAR binding, which would be unable to induce presynaptic differentiation. In alanine-scanning mutagenesis of the residues that are uniquely present in the ectodomain of SALM5 but not in other SALMs, we found that a double-alanine mutation of Ser329 and Ser360 (S329/360A) in the Ig domains of SALM5 significantly decreased SALM5 binding to LAR-Ecto-Fc (Fig. 5e,f). A similar result was obtained in cell aggregation assays (Fig. 5g,h). SALM5-S329/360A also showed weakened interactions with PTPδ and PTPσ in cell aggregation assays (Supplementary Fig. 6a–d).

Importantly, SALM5-S329/360A, but not wild-type (WT) SALM5, failed to induce synapsin I clustering in contacting axons in coculture assays (Fig. 5i,j). These results suggest that SALM5-dependent presynaptic differentiation is mediated by LAR-RPTPs. A control experiment in which the synapsin I clustering induced by SALM5 was normalized by tau, a marker of axonal fibers, to exclude the contribution of axon aggregation gave a similar result (data not shown).

We reasoned that SALM5-S329/360A, which lacks LAR binding but retains other domains intact, may exert a dominant-negative effect when overexpressed in neurons. Indeed, SALM5-S329/360A overexpressed in cultured hippocampal neurons reduced the density of synapsin I clusters along the dendrites (Supplementary Fig. 6e–g).

Lastly, when SALM5-expressing HEK293T cells were cocultured with hippocampal neurons infected with lentivirus carrying one of the three LAR-RPTP knockdown constructs^38^, PTPσ knockdown had apparently the strongest effect on SALM5-dependent synapsin I clustering whereas LAR or PTPδ knockdown had relatively small effects (Fig. 5k,l). In control experiments, the expression levels of LAR, PTPδ, and PTPσ mRNAs were similarly reduced by the individual knockdown constructs, down to ~20% of normal levels (Supplementary Fig. 7a). In addition, an shRNA-resistant PTPσ rescue construct could normalize SALM5-dependent synapsin I clustering to ~75% of original levels (Supplementary Fig. 7b,c), indicating that the effect of PTPσ knockdown is largely specific. These results collectively suggest that presynaptic LAR-RPTPs are important mediators of SALM5-induced presynaptic differentiation in the hippocampus.

### SALM5 regulates AMPAR-mediated synaptic transmission through LAR interaction

To explore the role of SALM5 in the regulation of synaptic function, we reduced the expression of SALM5 using shRNA transfection in organotypic hippocampal slice cultures (at DIV 3–4), followed by the measurement of excitatory synaptic transmission at Schaffer collateral (SC)-CA1 pyramidal synapses (at DIV 6–8).

**Figure 6:**
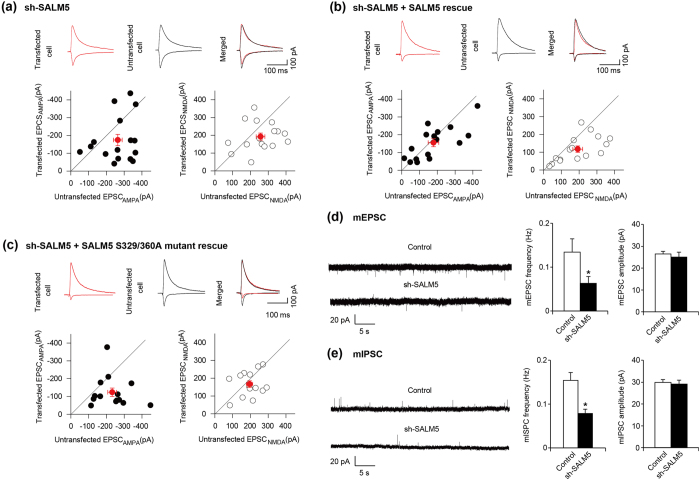
SALM5 regulates AMPAR EPSCs through mechanisms involving LAR interaction. (**a**) SALM5 knockdown reduces EPSC_AMPA_ in transfected neurons relative to untransfected neurons, whereas it has no effect on EPSC_NMDA_, although there was a strong tendency for a decrease (p = 0.052). *n* = 16. *p < 0.05 for EPSC_AMPA_, Student’s t-test. (**b**) Specificity of the SALM5 knockdown construct determined by the coexpression of the rescue construct. Neurons in slice cultures were transfected with sh-SALM5 + shRNA-resistant SALM5 (DIV 3–4), and EPSC_AMPA_ or EPSC_NMDA_ were measured at SC-CA1 synapses at DIV 6–8. *n* = 16. *p < 0.05 for EPSC_AMPA_ (SALM5 rescue), Student’s t-test. Note that the SALM5 rescue construct does not rescue the reduced EPSC_NMDA_, indicative of an off-target effect. (**c**) A mutant SALM5 (SALM5-S329/S360A) lacking LAR interaction fails to rescue SALM5-knockdown dependent suppression of EPSC_AMPA_. The experiments were performed as described in (**b**), expect that a mutant form of SALM5 was used. *n* = 16. *p < 0.05 for EPSC_AMPA_ (SALM5-S329/S360A rescue), Student’s t-test. (**d,e**) SALM5 knockdown suppresses the frequency but not amplitude of mEPSCs and mIPSCs in the CA1 pyramidal neurons of the hippocampus. Neurons in slice cultures were transfected with sh-SALM5 (DIV 3–4), and mEPSCs and mIPSCs were measured in CA1 neurons at DIV 6–8. *n* = 11 cells for mEPSC and 12 cells for mIPSC, *p < 0.05, Student’s t-test.

SALM5 knockdown in CA1 pyramidal neurons caused significant decrease in AMPAR-mediated excitatory postsynaptic currents (EPSC_AMPA_) relative to neighboring untransfected cells, although there was a strong tendency for a decrease of NMDA receptor (NMDAR)-mediated EPSCs (EPSC_NMDA_) (*p* = 0.052) (Fig. 6a).

Coexpression of shRNA-resistant rescue SALM5 together with the SALM5 knockdown construct rescued EPSC_AMPA_ (Fig. 6b), indicating that the knockdown construct is specific. However, the rescue construct failed to rescue the reduced EPSC_NMDA_, indicative of an off-target effect for NMDAR function. When a mutant SALM5 (S329/S360A) that lacks LAR binding was used in the rescue experiment, it failed to rescue EPSC_AMPA_ (Fig. 6c).

To gain further insights into the underlying mechanism, we knocked down SALM5 in hippocampal slice culture and found that SALM5 knockdown in CA1 pyramidal neurons substantially decreases the frequency but not amplitude of both miniature excitation postsynaptic currents (mEPSCs) and miniature inhibitory postsynaptic currents (mIPSCs) (Fig. 6d,e). These results collectively suggest that SALM5 regulates AMPAR-mediated synaptic transmission through mechanisms involving the interaction of postsynaptic SALM5 with presynaptic LAR-RPTPs, and that SALM5 regulates inhibitory synaptic transmission in addition to excitatory synaptic transmission, similar to the results from dissociated neurons^17^.

## Discussion

In the present study, we identified LAR-RPTPs as novel ligands of SALM5 that mediates SALM5-dependent presynaptic differentiation in a splicing-dependent manner.

Our data indicate that SALM5 interacts with all three known LAR-RPTPs—LAR, PTPδ, and PTPσ (Fig. 1). mRNAs for LAR, PTPδ, and PTPσ mRNAs display overlapping as well as differential distribution patterns in various brain regions^32^. In addition, SALM5 mRNAs are detected in various brain regions^14^,^16^, suggesting that their interactions may be widespread in the brain.

Pre- or postsynaptic localization of SALM5 and LAR at the ultrastructural level has not been determined due to the lack of suitable antibodies^17^,^30^. Previous and current results, however, suggest that postsynaptic SALM5 likely interacts with presynaptic LAR-RPTPs; 1) SALM5 expressed in heterologous cells or neurons induces presynaptic protein clustering, whereas SALM5 does not induce postsynaptic protein clustering^17^, and 2) presynaptic LAR-RPTPs interact with various postsynaptic adhesion molecules, including NGL-3, TrkC, IL1RAPL1, IL1RAcP, and Slitrks^30^,^32^,^33^,^34^,^35^,^36^,^37^,^38^. However, it is possible that SALM5 may be presynaptically localized and trans-synaptically interacts with postsynaptic SALM5 to regulate synapses, as supported by the reported transcellular, homophilic interaction of SALM5 in heterologous cells and cultured neurons^18^.

The Ig domains of LAR-RPTPs mediate SALM5 binding. This is similar to the reported Ig domain-dependent interactions of LAR-RPTPs with TrkC, IL1RAPL1, IL1RAcP, Slitrks, and SALM3^33^,^34^,^35^,^36^,^39^, but differs from the FN12-dependent interaction of LAR-RPTPs with NGL-3^30^,^32^. These results suggest that distinct domains of LAR-RPTPs mediate trans-synaptic interactions.

The meB splice insert strongly inhibits the interactions between LAR-RPTPs and SALM5 (Fig. 4 and Supplementary Fig. 4). This is similar to the reported meB-dependent inhibition of PTPσ binding to TrkC^33^, but contrasts with the meB-dependent interactions of LAR-RPTP with SALM3 and Slitrks^39^,^56^. It should be pointed out that the negative interaction between LAR and SALM3 in the present study (Fig. 1a–d) appears to be caused by the absence of the meB insert in our LAR constructs, as supported by the inhibition of SALM3-dependent presynaptic differentiation by LAR-Ecto-Fc containing the meB splice insert (Supplementary Fig. 5).

The meA splice had mixed influences on LAR-RPTP–SALM5 interactions, not affecting the LAR interaction, suppressing the PTPδ interaction, and both enhancing and suppressing the PTPσ interaction in a context-dependent manner. The positive regulation is reminiscent of meA-dependent interaction of PTPδ with IL1RAPL1 or IL1RAcP^35^,^36^, and the negative regulation is similar to the meB-dependent inhibition of PTPσ interaction with TrkC^33^. A similar bidirectional regulation has also been reported for neurexins; SS#4 (a 30-amino-acid insert at splice site #4) in neurexins enhances its interactions with Cbln1 and GluRδ2^62^, whereas it inhibits neurexin binding to LRRTM2^60^,^61^ and neuroligins^58^,^59^,^63^.

Our data implicate SALM5 in the regulation of AMPAR-mediated synaptic transmission. The AMPA-EPSC data suggest that SALM5 regulates AMPAR transmission through mechanisms involving the interaction of SALM5 with LAR-RPTPs. In addition, the mEPSC data, together with the known synaptogenic activities of SALM5^17^ and LAR-RPTPs^2^,^4^, suggests that SALM5 regulates AMPAR transmission through the modulation of the number but not strength of individual excitatory synapses. These modulations likely involve postsynaptic SALM5 because the slice knockdown of SALM5 occurs in CA1 pyramidal postsynaptic neurons. In addition, it is likely to involve presynaptic LAR-RPTPs because the frequency but not amplitude of mEPSCs was decreased. However, postsynaptic LAR-RPTPs have been suggested to play important roles in the development and maintenance of excitatory synapses, as supported by, for example, the decreases in both frequency and amplitude of mEPSCs by the inhibition of postsynaptic LAR-RPTPs^28^. Therefore, whether postsynaptic or even presynaptic SALM5 interacts with postsynaptic LAR-RPTPs to regulate AMPARs remains to be determined.

Lastly, SALM5/Lrfn5 has been implicated in ASDs and schizophrenia^19^,^20^,^21^,^22^,^24^,^25^. In addition, PTPδ is implicated in ASDs, ADHD, bipolar disorder, and restless leg syndrome^51^,^52^,^53^,^54^,^55^,^65^. Therefore, our findings might eventually help us understand the mechanisms underlying SALM5- and LAR-RPTP-related brain dysfunctions.

In conclusion, we have identified LAR-RPTPs as novel and splicing-dependent ligands that mediate SALM5-dependent presynaptic differentiation. In addition, SALM5 regulates AMPAR-mediated synaptic transmission through mechanisms involving the interaction with LAR-RPTPs. These results suggest that SALM5 contributes to synapse development and maintenance.

## Materials and Methods

### DNA constructs and antibodies

For Myc tagging of SALMs, the epitope was inserted between aa 16 and 17 of mouse SALM3 and aa 19 and 20 of mouse SALM5, respectively, in pGW1 (British Biotechnology). The Myc epitope of SALM5-Ecto-pDis was replaced with stop codon to generate N-terminally HA-tagged soluble SALM5-Ecto. For LAR-IgFN14-Fc, Ig1-3 + FN1-4 of LAR (aa 1-699) were subcloned into pFc-N1 (modified pEGFP-N1 in which EGFP was replaced with human Fc). The following LAR-RPTP deletion variants were subcloned into pDisplay (Invitrogen): human LAR-Ig (aa 17–308), human LAR-Ig-A^−^B^+^ and LAR-Ig- A^+^B^+^ (aa 30–318), human PTPδ-Ig (aa 21–309), human PTPσ-Ig (aa 30–318). For LAR-Ig-Fc, the signal peptide of Ig-kappa and the Ig domains of LAR were subcloned into pFc-N1. Myc-SALM5 deletion variants were subcloned region into GW1: Myc-SALM5-ΔLRR (Δaa 20–285), Myc-SALM5-ΔIg (Δaa 299–364), Myc-SALM5-ΔFNIII (Δaa 408–495). Splice variants of the Ig domains of LAR-RPTPs were subcloned into pDisplay. Full-length mouse PTPσ, PTPδ, and LAR have been previously described: PTPσ-A^−^B^−^-CFP, PTPσ-A^+^B^−^-CFP, PTPσ-A^−^B^+^-CFP, PTPσ-A^+^B^+^-CFP^33^, PTPδ-A^−^B^−^, PTPδ-A^+^B^−^, PTPδ-A^−^B^+^, PTPδ-A^+^B^+^, LAR- A^−^B^−^, LAR- A^+^B^−^, LAR- A^−^B^+^, and LAR- A^+^B^+^ in pcDNA3 vector^35^. Point mutants of SALM5-Ecto-pDis, untagged SALM5 in pGW1, and SALM5 in pIRES were generated using the Quickchange kit (Stratagene). The following constructs have been described previously: LAR-Ecto-pDis, LAR-FNIII-pDis^32^, SALMs 1-5-Ecto-pDis, untagged SALM5, sh-SALM5^17^, HA-CD8^14^, LAR-Ig-A^−^B^−^-pDis, LAR-Ig-A^+^B^−^-pDis^56^, NGL-3-EGFP, LAR-ECFP, LAR-Ecto-Fc^30^, shRNA lentiviral expression constructs for LAR-RPTPs^38^, and shRNA-resistant SALM5 (wild-type; pIRES-SALM5)^17^. The following antibodies have been described previously: EGFP (1431)^30^, SALM5 (1907)^17^, and Shank2^66^. The following antibodies were purchased: Myc, HA (Santa Cruz), synapsin I (Millipore), and α-tubulin (Sigma).

### Cell aggregation assay

Cell aggregation assays were performed as described^32^. Briefly, two L-cell groups were transfected with EGFP + SALMs-Ecto-pDis, or RFP (DsRed) + LAR-Ecto-pDis. After 24 hours, L cells were trypsinized and resuspended in DMEM, followed by L cell mixing and rotation at room temperature for 2 hours for aggregation. Areas of cell aggregates in pixels were calculated using MetaMorph, and those that fall below of the size of an average single cell were removed and used to calculate an average aggregate size in arbitrary units (AU).

### Soluble LAR-binding assay

Purified soluble LAR-Ecto-A^−^B^−^-Fc (10 μg/ml) was added to SALM-expressing live HEK293T cells and incubated for 2 hours. Without permeabilization, cells were fixed with 4% paraformaldehyde/4% sucrose and incubated with antibodies. For quantitative LAR-binding assays, SALM5-expressing HEK293T cells were transferred to 96-well plates and grown for 24 hours. After fixation in 4% paraformaldehyde/4% sucrose, cells were incubated with increasing concentrations of LAR-Ecto-Fc for 1 hour, followed by incubation with HRP-conjugated anti-human-Fc antibodies (Sigma) and color reaction with 3,3′,5,5′-Tetramethylbenzidine (Sigma). Integrated intensities of bound proteins in a field of view were normalized by those of the proteins expressed in the cells.

### Recombinant protein expression and purification

The Ig1-3-FNIII12 domain (aa 27A–513A), Ig1-3-A^+^B^+^ (aa 30D-318L), and FN12 (aa 312Q–510G) of human LAR were cloned into pAcGP67 (BD Bioscience), modified for C-terminal protein-A tagging. Since human SALM5 was hardly expressed in High Five insect cells (Invitrogen), hybrid LRR techniques were used to generate a chimeric hySALM5 proteins^67^. Briefly, the N terminus of VLRB61 (1M–82L) was combined with the LRR and Ig domains of human SALM5 (aa 59A–374I) by overlap PCR, and the resulting chimeric gene for hySALM5 was cloned into pVL1393 (Invitrogen), modified for C-terminal Fc tagging. All constructs described above contain a thrombin cleavage site (LVPRGS) between target proteins and tags. The High Five insect cells were transfected with corresponding P4 baculovirus for 3 days and harvested. The supernatants containing secreted proteins were loaded onto IgG sepharose column to purify protein A-fused proteins, or onto protein A sepharose column to purify Fc-tagged protein. The protein A or Fc tags were thrombin cleaved (0.5% v/v) for 16 hours at 4 °C, followed by gel-filtration purification using Superdex 200 (GE Healthcate Life Science).

### Biolayer interferometry (BLI)

For in vitro binding assays using the BLItz system (ForteBio), purified proteins were prepared in low salt buffer (20 mM HEPES, 50 mM NaCl, pH 7.0). Prior to the assay, protein A biosensors were hydrated in MilliQ for 10 min, followed by equilibration in a low salt buffer for 60 s. 10 μM of LAR-Ig-A^−^B^−^ (Ig1-3)-Fc, LAR-FN12 (FNIII1-2)-Fc, or hySALM5-Ecto (LRR + Ig-A^−^B^−^)-Fc was immobilized to the equilibrated sensors for 120 s. After dissociation, the primed biosensors were used to analyse the interaction between immobilized proteins and analytes. Three different analyte concentrations were used with the same primed biosensors. For LAR-Ig-FN12, which could not be purified, hySALM5-Ecto-Fc was pre-bounded to the equilibrated sensors. At the end of each association-dissociation steps, a high salt buffer (20 mM HEPES, 1M NaCl, pH 7.0) was used to wash off analytes from the primed biosensors. Affinity constants were determined using the global fitting function in the advanced kinetic program of BLItz.

### Mixed-culture assay

Mixed-culture assays were performed as described^68^. Briefly, primary hippocampal neuron cultures at DIV 9/10 prepared from E18–19 rat hippocampi were cocultured with HEK293T cells expressing SALMs/CD8, followed by immunostaining at DIV 12/13. Hippocampal neurons were transfected for three days prior to coculture. For competitive inhibition, Fc alone, LAR-Ecto-A^−^B^−^-Fc, or LAR-Ig-A^+^B^+^-Fc (10 μg/ml), were added to neuron-HEK293T cocultures for 3 days (DIV 9/10–12/13). Mixed-culture assays under lentiviral knockdown conditions were performed with as described^60^. Briefly, cultured hippocampal neurons were infected at DIV3–4 with the lentiviruses expressing shRNAs for LAR, PTPδ, or PTPσ^38^, and incubated until HEK293T cells transfected with EGFP, or pDisplay-LAR, for 48 hours were added at DIV 10. At DIV13, the cells were double-immunostained for EGFP and synapsin. Levels of knockdowns were determined in parallel by quantitative RT-PCR. For image quantification, fluorescence intensity of synapsin I puncta was normalized to cell area using MetaMorph (Molecular Device).

### Lentivirus production

To generate the recombinant lentiviruses, human embryonic kidney 293T cells were transfected with three plasmids—L-309 vectors (L-309 LAR KD, L-309 PTPδ KD, L-309 PTPσ, or L-309 alone), or L-313 PTPσ-A^−^B^−^, PAX2, and pMD2G—using FuGENE-6 (Roche), as previously described^69^.

### Transfection of neurons and immunocytochemistry

Cultured hippocampal neurons were transfected using mammalian transfection kit (Clontech), or lipofectamine LTX and PlusTM Reagent (Invitrogen), fixed with 4% paraformaldehyde/4% sucrose, and permeabilized with 0.2% Triton x-100 in phosphate buffered saline, followed by incubation with primary antibodies and Cy3-, Cy5-, or FITC-conjugated secondary antibodies (Jackson ImmunoResearch).

### Image acquisition and quantification

All z-stacked images were randomly acquired by confocal microscopy (LSM510 and LSM780, Zeiss). For quantification of cell aggregation and Fc-binding assays, each field of view was counted as n of 1. For coculture assays and electrophysiology experiments, each cell or neuron was counted as n of 1. The results presented in all figures are from one set of experiments, although essentially the same conclusions were obtained from additional independent experiments (mostly once or twice more) with the exception of electrophysiology (Fig. 6) for which data from two to three independent experiments were pooled.

### Slice electrophysiology

Hippocampal slices (350 μm; 6–8-day-old Wistar rat; McIlwain tissue chopper) were cultured on semipermeable membrane inserts (Millipore) in a six-well plate containing culture medium (78.8% MEM, 20% heat-inactivated horse serum, 25 mm HEPES, 10 mM D-glucose, 26 mM NaHCO_3_, 2 mM CaCl_2_, 2 mM MgSO_4_, 0.0048% 25% Ascorbic Acid, 0.1% 1 mg/ml Insulin, pH 7.3, 320–330 mOsm). Slices were cultured for DIV 6–8 with changes of media without antibiotics every 2 days. Neurons were transfected using a biolistic gene gun (Helios Gene-gun system) at DIV3–4 (100 μg DNA; 90% test constructs; 10% pEGFP-C1). Electrophysiological recordings were performed at 3–4 days after transfection.

For whole-cell patch recordings, cultures were superfused with a warmed (28°–29°C) recording solution which comprised: (mM) NaCl, 119; KCl, 2.5; NaHCO_3_, 26; NaH_2_PO_4_, 1; MgCl_2_, 4; D-glucose, 11; CaCl_2_, 4 with added 10 μM chloro adenosine and 20 μM picrotoxin. A stimulating electrode was placed in SC-CA1 input, and neighbouring transfected and untransfected neurons were whole-cell patch clamped. Every 15 s, stimuli were delivered to the electrode (0.033 Hz). EPSC_AMPA_ was estimated as the peak EPSC amplitude at a holding potential of −70 mV, and EPSC_NMDA_ was estimated at a holding potential of +40 mV measured 80−90 msec after the peak EPSC_AMPA_. Only cells that had an initial *R*_s_ (series resistance) <20 MΩ with <20% changes during recording were included in data analysis. mEPSCs were recorded in the presence of 20 μM picrotoxin, 1 μM bicuculline (Tocris) and 0.5 μM tetrodotoxin (Abcam). mIPSCs were recorded in the presence of 10 μM CNQX (HelloBio), 50 μM AP5 (HelloBio), and 0.5 μM tetrodotoxin. mEPSCs (holding potential, −70 mV) and mIPSCs (holding potential, 0 mV) were analyzed by Mini Analysis Software (Synaptosoft), with minimum event detection threshold values of 15 pA and 20 pA for mEPSCs and mIPSCs, respectively.

## Additional Information

**How to cite this article**: Choi, Y. *et al.* SALM5 trans-synaptically interacts with LAR-RPTPs in a splicing-dependent manner to regulate synapse development. *Sci. Rep.* **6**, 26676; doi: 10.1038/srep26676 (2016).

## Supplementary Material

Supplementary Information
