# Supplementary material for: SALM5 trans-synaptically interacts with LAR-RPTPs in a splicing-dependent manner to regulate synapse development
Source: Sci Rep. 2016 May 26;6:26676. doi: 10.1038/srep26676 (PMC4881023; doi:10.1038/srep26676)
Supplement: Supplementary Information [file srep26676-s1.pdf]

SALM5 trans-synaptically interacts with LAR-RPTPs in a splicing-dependent manner to regulate synapse development

Yeonsoo Choi,[1,#] Jungyong Nam,[1,#] Daniel J Whitcomb,[2,#] Yoo Sung Song,[3,#] Doyoun Kim,[4] Sangmin Jeon,[5] Ji Won Um,[5,6] Seong-Gyu Lee,[1] Jooyeon Woo,[1] Seok-Kyu Kwon,[1] Yan Li,[4] Won Mah,[7] Ho Min Kim,[9] Jaewon Ko,[5] Kwangwook Cho,[2,8,*] and Eunjoon Kim[1,4,*]

[1]Department of Biological Sciences, Korea Advanced Institute for Science and Technology (KAIST), Daejeon 305-701, Korea; [2]Henry Wellcome Laboratories for Integrative Neuroscience and Endocrinology, School of Clinical Sciences, Faculty of Health Sciences, University of Bristol, Whitson Street, Bristol BS1 3NY, United Kingdom; [3]Department of Nuclear Medicine, Seoul National University Bundang Hospital, Gyeonggi-do, 463–707, Korea; [4]Center for Synaptic Brain Dysfunctions, Institute for Basic Science (IBS), Daejeon 305-701, Korea; [5]Department of Biochemistry, College of Life Science and Biotechnology, Yonsei University, Seoul 120-749, Korea; [6] Department of Physiology and BK21 PLUS Project for Medical Science, Yonsei University College of Medicine, Seoul 120-752, Korea; [7]Department of Anatomy and Neurobiology, School of Dentistry, Kyungpook National University, Daegu, Korea; [8]Centre for Synaptic Plasticity, University of Bristol, Bristol BS1 3NY, United Kingdom; United Kingdom; [9]Graduate School of Medical Science and Engineering, KAIST, Daejeon 305-701, Korea; [#]These authors contributed equally to the work; *Co-corresponding authors.

Correspondence should be addressed to
Eunjoon Kim
Center for Synaptic Brain Dysfunctions, Institute for Basic Science (IBS), Daejeon 305-701, Korea and Department of Biological Sciences, Korea Advanced Institute of Science and Technology (KAIST), Daejeon 305-701, Korea
E-mail: kime@kaist.ac.kr, Tel: +82-42-350-2633, Fax: +82-42-350-8127

Kwangwook Cho
Henry Wellcome Laboratories for Integrative Neuroscience and Endocrinology, Centre for Synaptic Plasticity, School of Clinical Sciences, University of Bristol, Whitson Street, Bristol BS1 3NY, United Kingdom
E-mail:Kei.Cho@bristol.ac.uk, Tel:+44-117-331-3048, Fax: +44-117-331-3046.

Supplementary figure

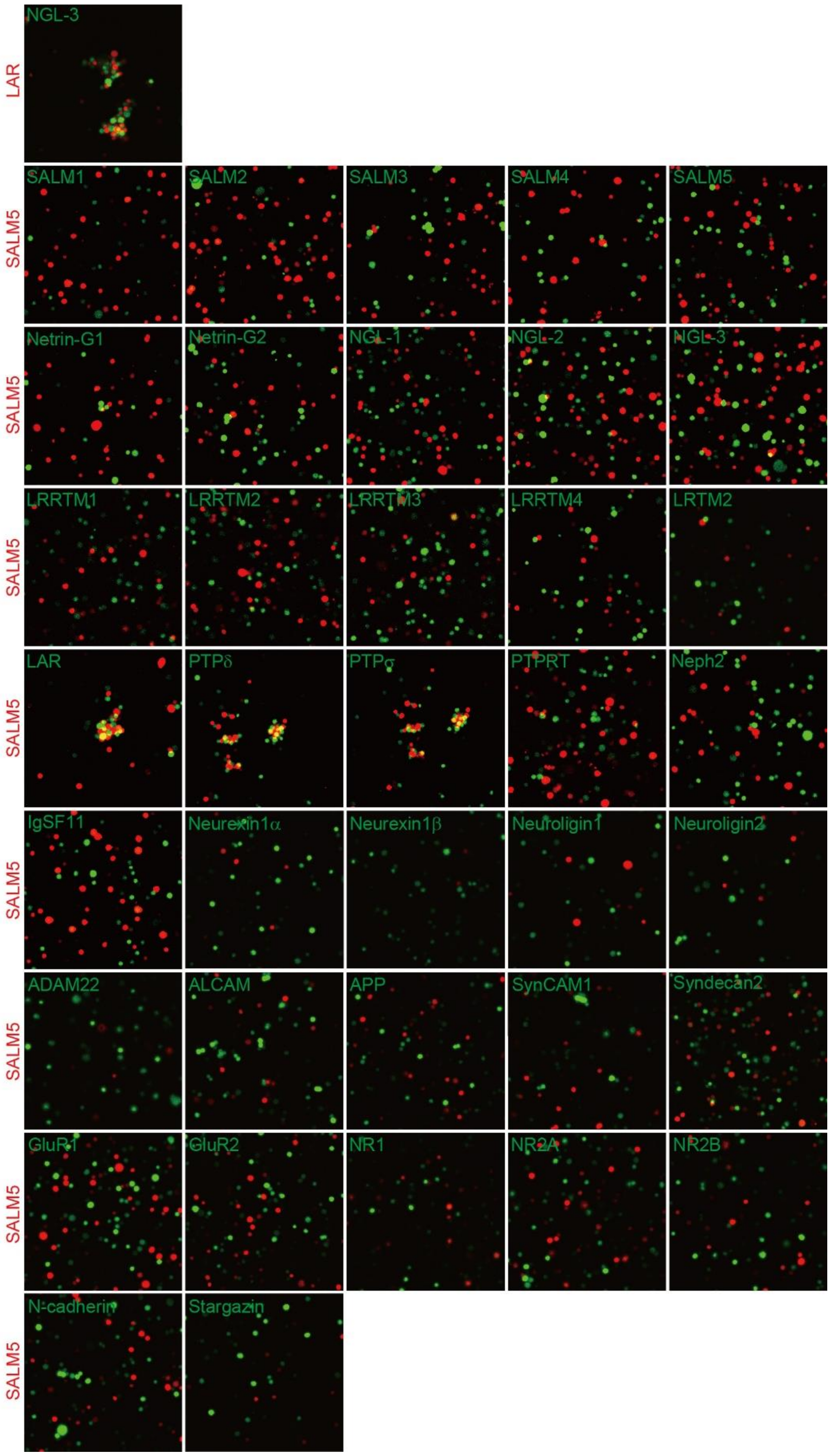

Supplementary Figure 1. Synaptic membrane proteins used to test the interaction with SALM5 in cell aggregation assays.

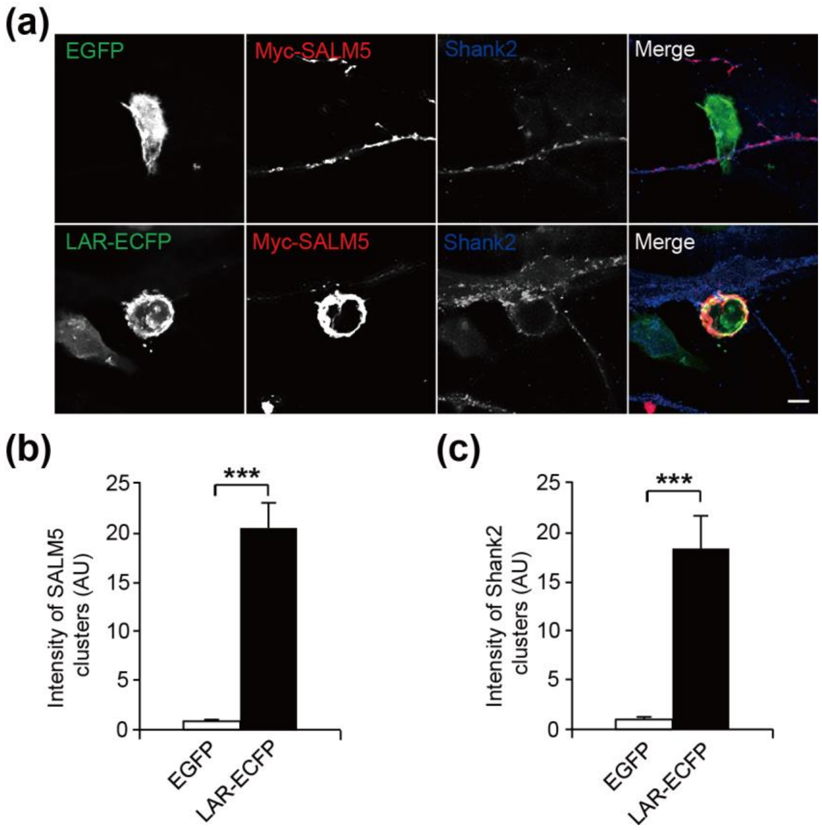

Supplementary Figure 2. **LAR-expressing HEK293T cells induce Shank2 (a postsynaptic scaffold) clustering in contacting neurites of cocultured neurons**. (a) HEK293T cells expressing LAR (C-terminal ECFP tag) were cocultured with hippocampal neurons (DIV 14–17) transfected with SALM5 (N-terminal Myc tag; DIV 12–14) and stained for EGFP (for LAR), Myc (for SALM5), and endogenous Shank2. (b and c) For quantification, intensities of SALM5 or Shank2 were normalized to HEK cell areas. Scale bar, 10 μm.

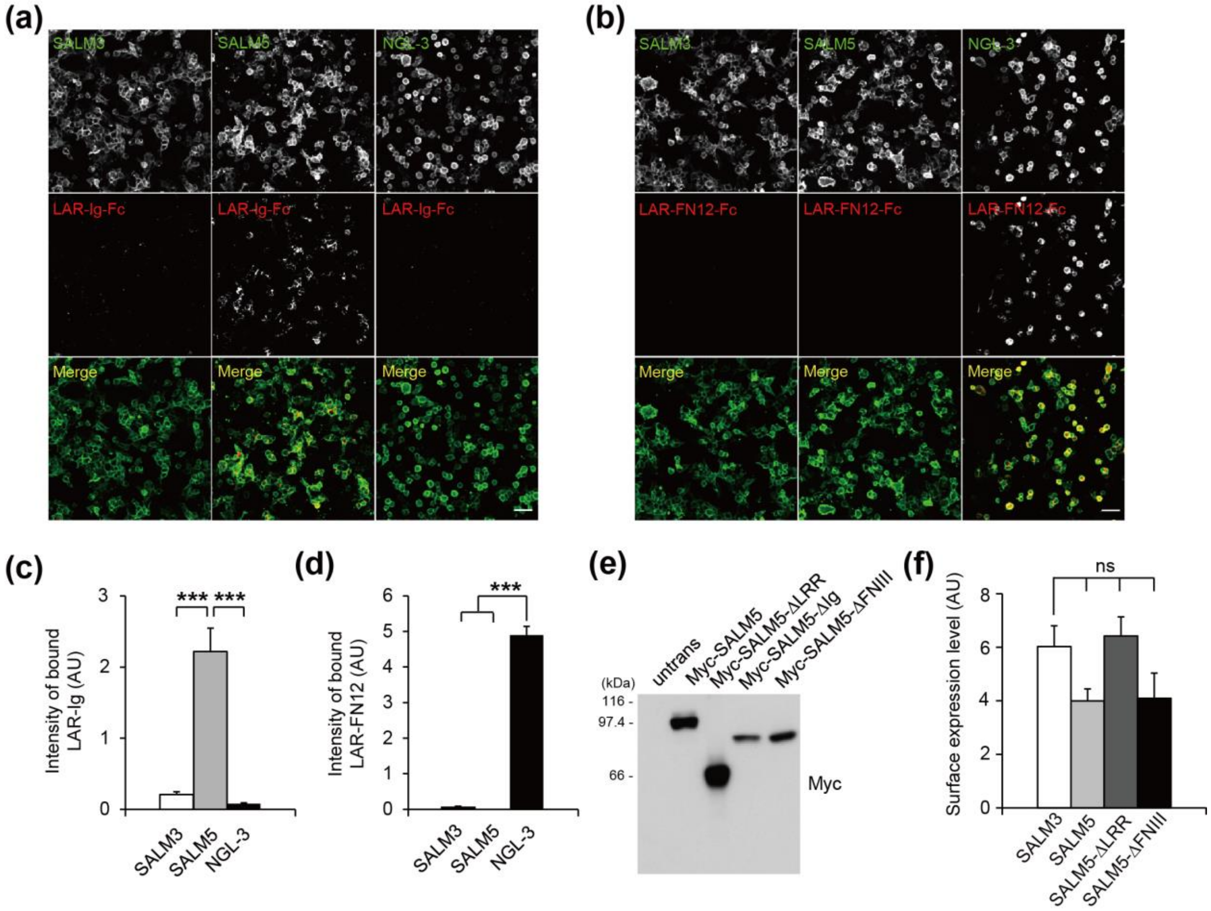

Supplementary Figure 3. **LAR binds to SALM5 and NGL-3 via its Ig and FNIII domains, respectively, in soluble protein binding assays.**
(a) Purified proteins of LAR-Ig (Ig domains only) fused to Fc (LAR-Ig-Fc) bind to HEK293T cells expressing SALM5 but not to those expressing SALM3 or NGL-3.
(b) Purified proteins of LAR-FN12-Fc (first two FNIII domains only) bind to HEK293T cells expressing NGL-3 but not to SALM3 or SALM5.
(c and d) Quantification of the results in (a) and (b). Mean ± s.e.m. $n = 5$ fields of view, ***$p < 0.001$, ANOVA-Tukey's test.
(e) Immunoblot analysis of SALM5 deletion variants. HEK293T cells were transfected with full-length and deletion variants of Myc-tagged SALM5, and cell lysates were analyzed by immunoblotting with Myc antibodies.
(f) Quantification of the results in **Fig. 2d,e** to determine the surface expression levels of SALM5 deletions variants, revealed by Myc staining without permeabilization for surface Myc-SALM5 expressed in HEK293T cells. $n = 10$ fields of view, ns, not significant, ANOVA-Tukey's test.

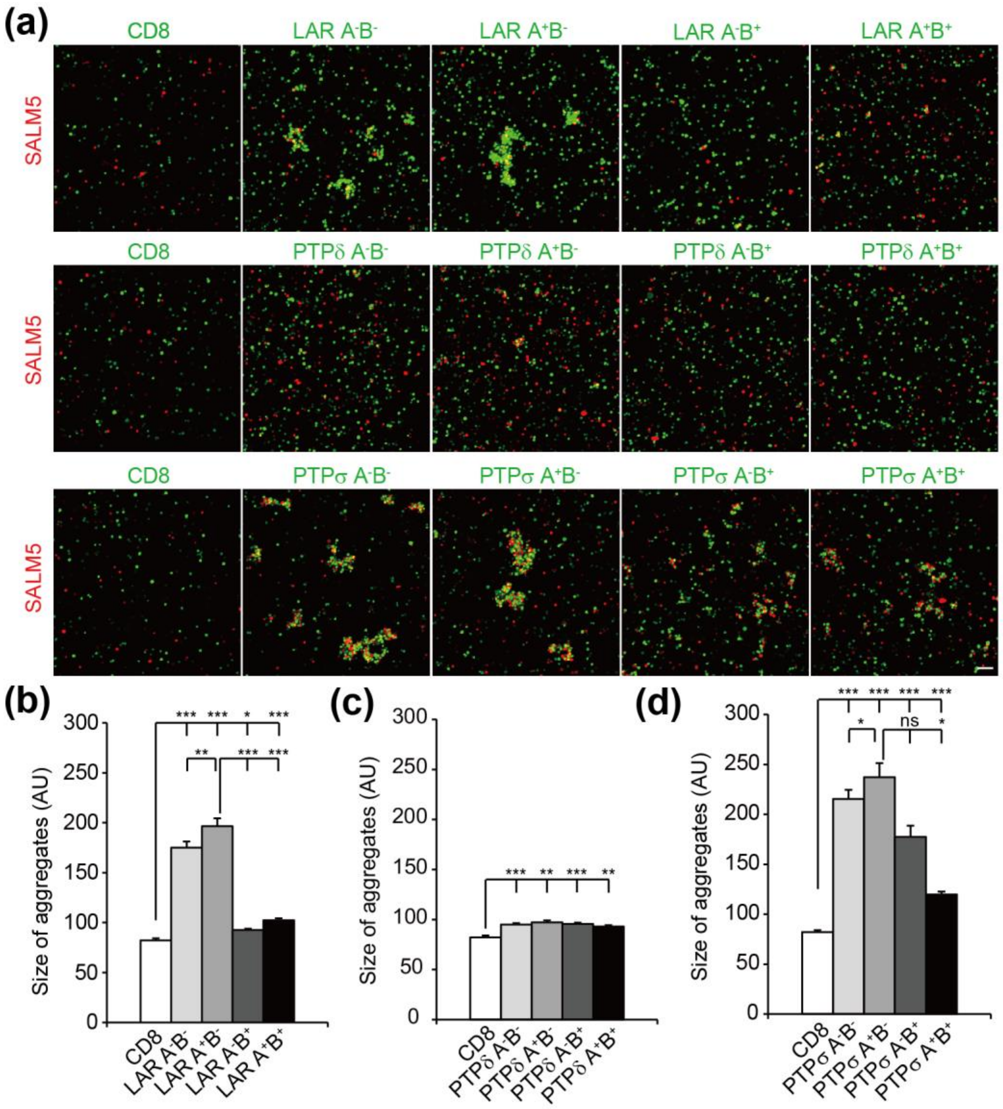

Supplementary Figure 4. Splice inserts in full-length LAR, PTPδ, and PTPσ differentially regulate SALM5 binding.

(a) Representative images of cell aggregation between SALM5 and full-length LAR-RPTPs with or without meA and meB splice inserts. The relatively weak cell aggregations observed in PTPδ appears to be due to low expression levels of full-length PTPδ, relative to Ig domain-only PTPδ described in **Fig. 3**. Scale bar, 100 μm. (b-d) Quantification of the results in (a). Mean ± s.e.m. n = 16 fields of view for CD8, 28 for LAR A^-B^-, 34 for LAR A^+B^-, 21 for LAR A^-B^+, 19 for LAR A^+B^+, 17 for PTPδ A^-B^-, 17 for PTPδ A^+B^-, 15 for PTPδ A^-B^+, 17 for PTPδ A^+B^+, 39 for PTPσ A^-B^-, 49 for PTPσ A^+B^-, 33 for PTPσ A^-B^+, and 34 for PTPσ A^+B^+, *$p < 0.05$, **$p < 0.01$, ***$p < 0.001$, ns, not significant, Kruskal-Wallis test with Dunn's multiple comparison posthoc test.

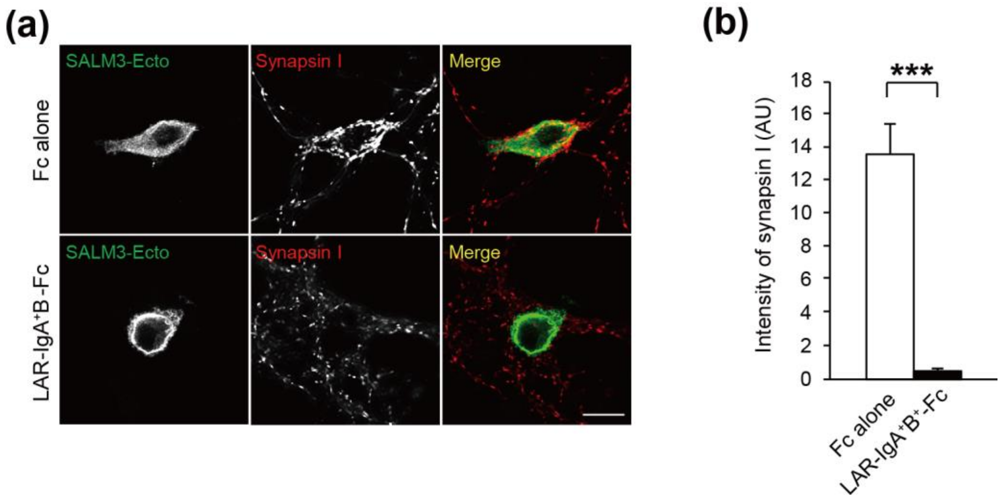

Supplementary Figure 5. Soluble LAR with meB inhibits SALM3-dependent synapsin I clustering.

SALM3-expressing HEK293T cells were cocultured with hippocampal neurons in the presence of LAR-Ig-$A^{+}B^{+}$-Fc, or Fc alone, followed by immunostaining for synapsin I and SALM3 (Myc). Scale bar, 15 μm.

(b) Quantification of the results in (a). Mean ± s.e.m. n = 33 fields of view for Fc and LAR-Ecto-Fc, ***p < 0.001, Mann Whitney test.

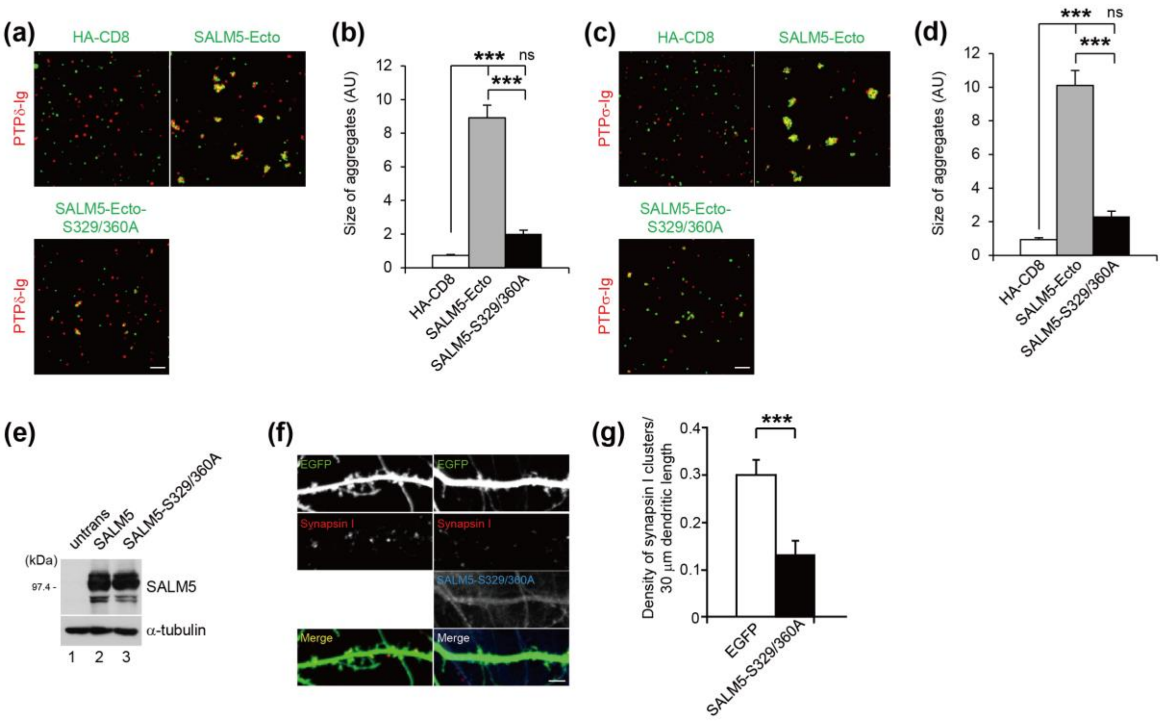

Supplementary Figure 6. SALM5-S329/360A shows reduced binding to PTPδ and PTPσ in cell aggregation assays, and causes a reduction in synapsin I intensity when overexpressed in neurons.

(a-d) SALM5-S329/360A shows reduced binding to PTPδ and PTPσ in cell aggregation assays. L cells expressing SALM5-Ecto-pDis (WT or S329/360A) were mixed with a separate group of L cells expressing PTPδ-Ig-pDis or PTPσ-Ig-pDis to assay for cell aggregation. HA-CD8 was used as a negative control. Mean ± s.e.m. n = 10 fields of view for PTPδ and PTPσ, ***$p < 0.001$, ANOVA-Tukey's test. Scale bar, 100 μm.

(e-g) Overexpression of SALM5-S329/360A in cultured hippocampal neurons (DIV 12-15) causes a decrease in the density of synapsin I clusters in dendrites. Mean ± s.e.m. n = 12 neurons, ***$p < 0.001$, Student's t-test. Scale bar, 5 μm.

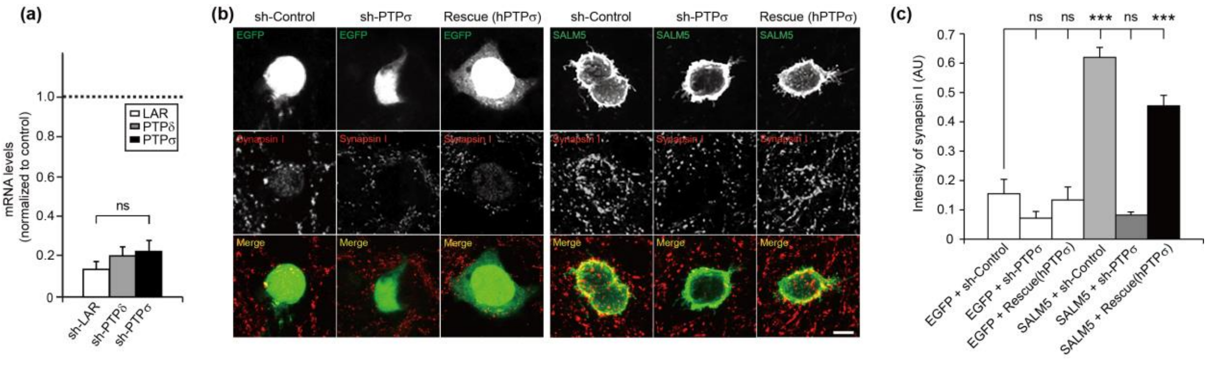

Supplementary Figure 7. Characterization of lentiviral knockdown constructs for LAR, PTPδ, and PTPσ.

(a) Normalized levels of LAR, PTPδ, and PTPσ mRNAs in cultured hippocampal neurons infected with the indicated knockdown constructs (DIV 10-13) were characterized by quantitative RT-PCRs. *n* = 3 cultures for sh-LAR, sh-PTPδ, and sh-PTPσ. Scale bar, 10 μm.

(b) Deficit in SALM5-mediated synaptogenic activity in PTPσ-deficient neurons is restored by re-expression of full-length PTPσ. Hippocampal neurons infected at DIV3 with lentiviruses expressing sh-control, sh-PTPσ, or coinfected with sh-PTPσ and PTPσ (MeA-, MeB-) were cocultured for 3 days (DIV10–13) with HEK293T cells expressing EGFP alone (Control) or EGFP+SALM5-pDis (SALM5), followed by staining for EGFP and synapsin I, Scale bar, 10 µm.

(c) Quantification of the results in (b); synapsin I signals were normalized to EGFP fluorescence. *n* = 10 HEK293T cells for EGFP/sh-Control, 12 for EGFP/sh-PTPσ, 10 for EGFP/Rescue, 14 for SALM5/sh-Control, 10 for SALM5/sh-PTPσ, and 14 for SALM5/Rescue. ***$p < 0.001$, ANOVA-Tukey's test.
